# Supplementary material for: Global analyses of biosynthetic gene clusters in phytobiomes reveal strong phylogenetic conservation of terpenes and aryl polyenes
Source: mSystems. 2023 Jul 6;8(4):e00387-23. doi: 10.1128/msystems.00387-23 (PMC10469690; doi:10.1128/msystems.00387-23)
Supplement: Supplemental figures — Figures S1 to S3. [file msystems.00387-23-s0001.pdf]

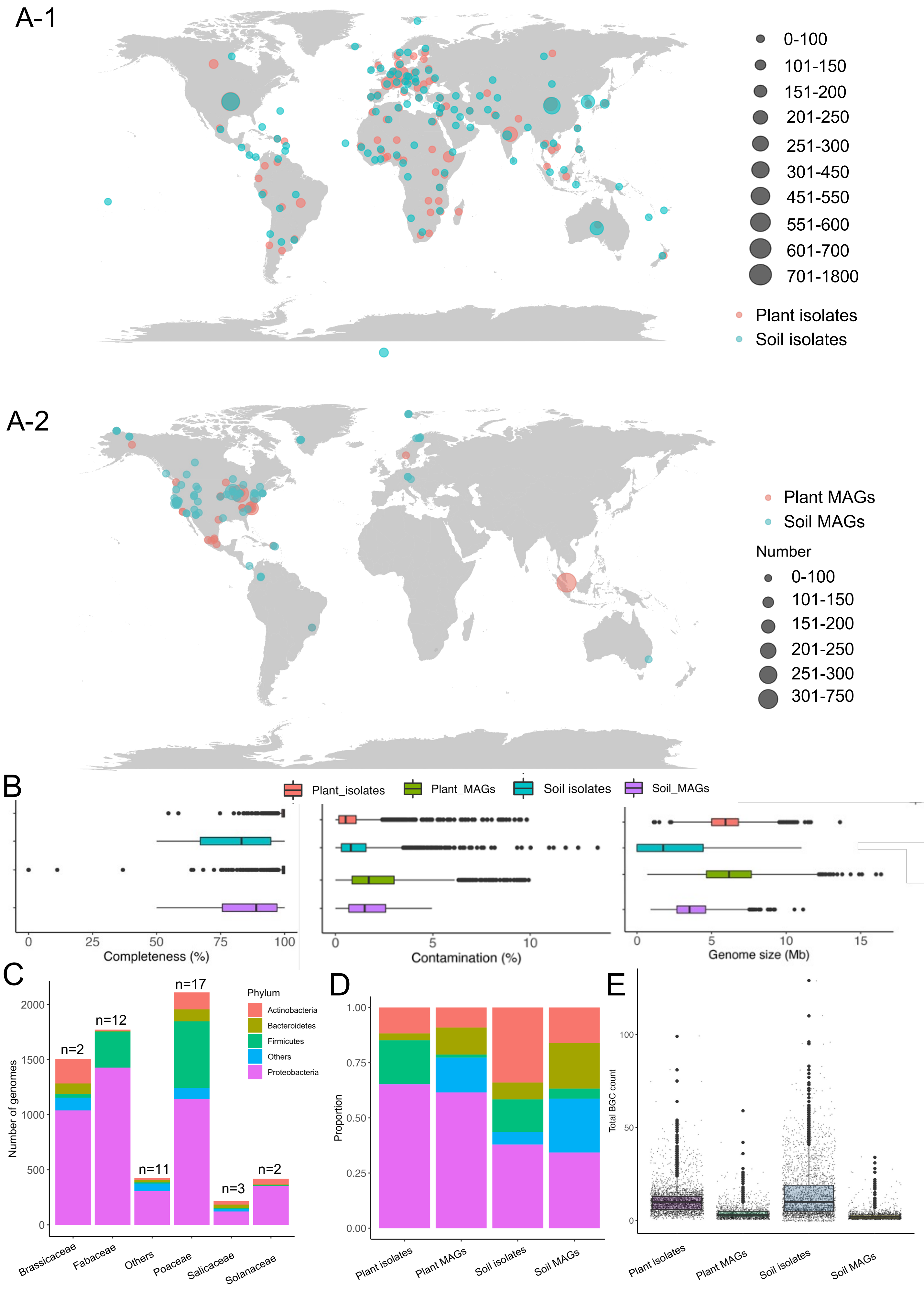

A-1
0-100
101-150
151-200
201-250
251-300
301-450
451-550
551-600
601-700
701-1800
Plant isolates
Soil isolates
A-2
Plant MAGs
Soil MAGs
Number
0-100
101-150
151-200
201-250
251-300
301-750
B
Plant_isolates
Plant_MAGs
Soil isolates
Soil_MAGs
Completeness (%)
Contamination (%)
Genome size (Mb)
C
Phylum
Actinobacteria
Bacteroidetes
Firmicutes
Others
Proteobacteria
Number of genomes
n=2
n=12
n=11
n=17
n=3
n=2
Brassicaceae
Fabaceae
Others
Poaceae
Salicaceae
Solanaceae
D
Proportion
Plant isolates
Plant MAGs
Soil isolates
Soil MAGs
E
Total BGC count
Plant isolates
Plant MAGs
Soil isolates
Soil MAGs

Figure S1. Metadata information and quality profile of isolates and MAGs

A.Global distribution of the genomes of isolates and MAGs, included in this study. The size of the circles indicate the number of genomes and colors represent either MAGs or isolates. Inset A-1 and A-2 are presented for isolates and MAGs, respectively. **B.** Completeness, contamination, and size profile of genomes included in this study. **C.** Host plant family level distribution of plant associated genomes (both isolates, and MAGs combined) included in this study. The phylum-level distribution of these genomes is shown in different colors. The numbers on top of each bar indicate the number of representative plant species under each plant family. **D.** The microbiome composition of plant isolates, soil isolates, plant MAGs, and soil MAGs datasets at the phylum level. **E.** Total BGC count per genome among members of plant isolates, soil isolates, plant MAGs, and soil MAGs.

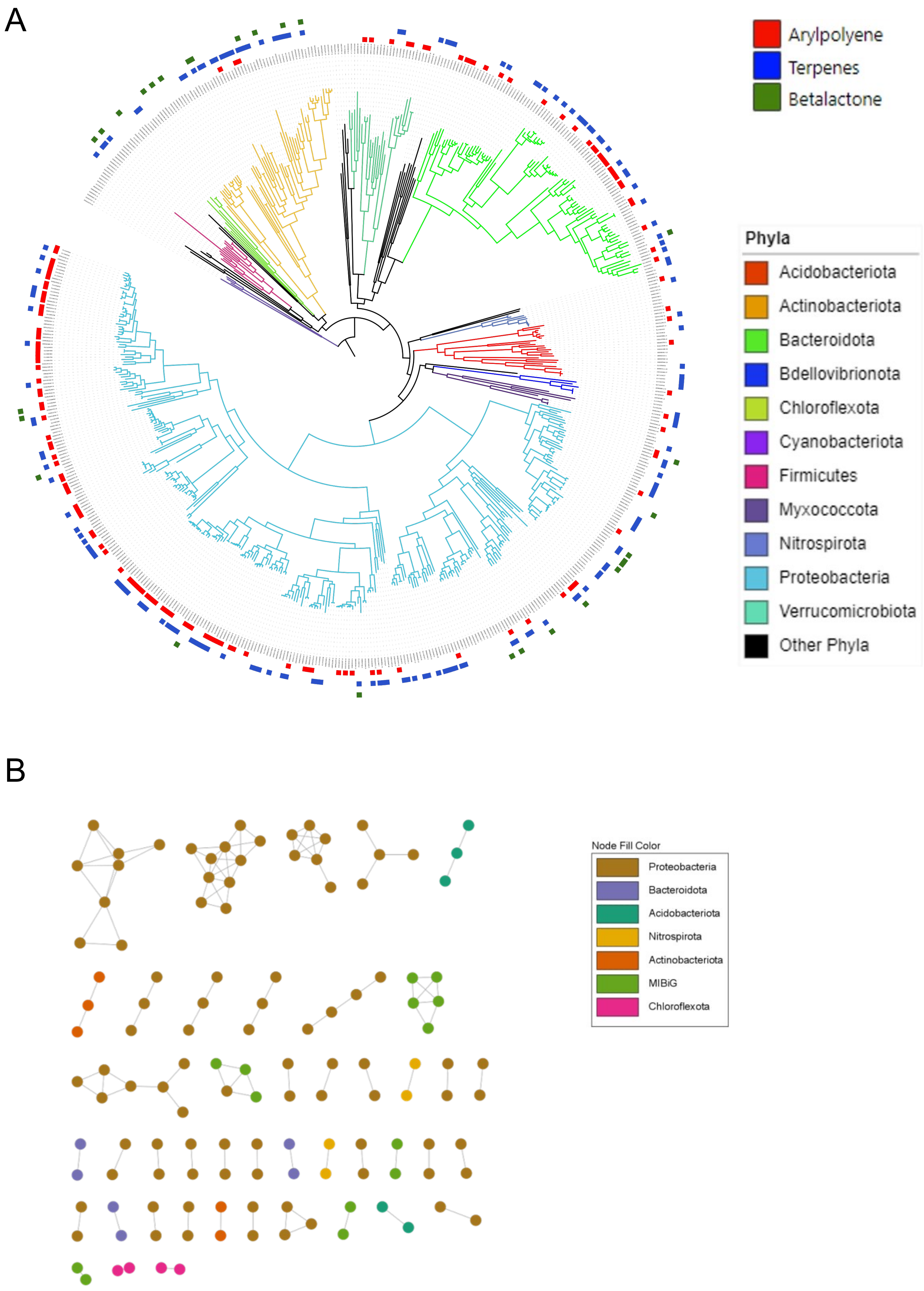

A
Arylpolyene
Terpenes
Betalactone
Phyla
Acidobacteriota
Actinobacteriota
Bacteroidota
Bdellovibrionota
Chloroflexota
Cyanobacteriota
Firmicutes
Myxococcota
Nitrospirota
Proteobacteria
Verrucomicrobiota
Other Phyla
B
Node Fill Color
Proteobacteria
Bacteroidota
Acidobacteriota
Nitrospirota
Actinobacteriota
MIBiG
Chloroflexota

Figure S2. Conservation patterns of terpenes and aryl polyenes in plant MAGs dataset.

A.Phylogenetic tree showing presence-absence pattern of terpenes, aryl polyenes and betalactones in plant MAGs. Stronger phylogenetic conservation of terpenes and aryl polyenes compared to betalactones are shown by their three respective outer rings (blue, red, and green respectively). The taxonomic classes of the plant MAGs are shown in different colors of the phylogenetic tree branches. **B.** Sequence similarity network of terpenes GCFs found in plant MAGs (singletons are not shown). Each node represents individual terpene gene clusters, colored according to taxonomy. Nodes with existing reference (MIBiG) are highlighted in green.

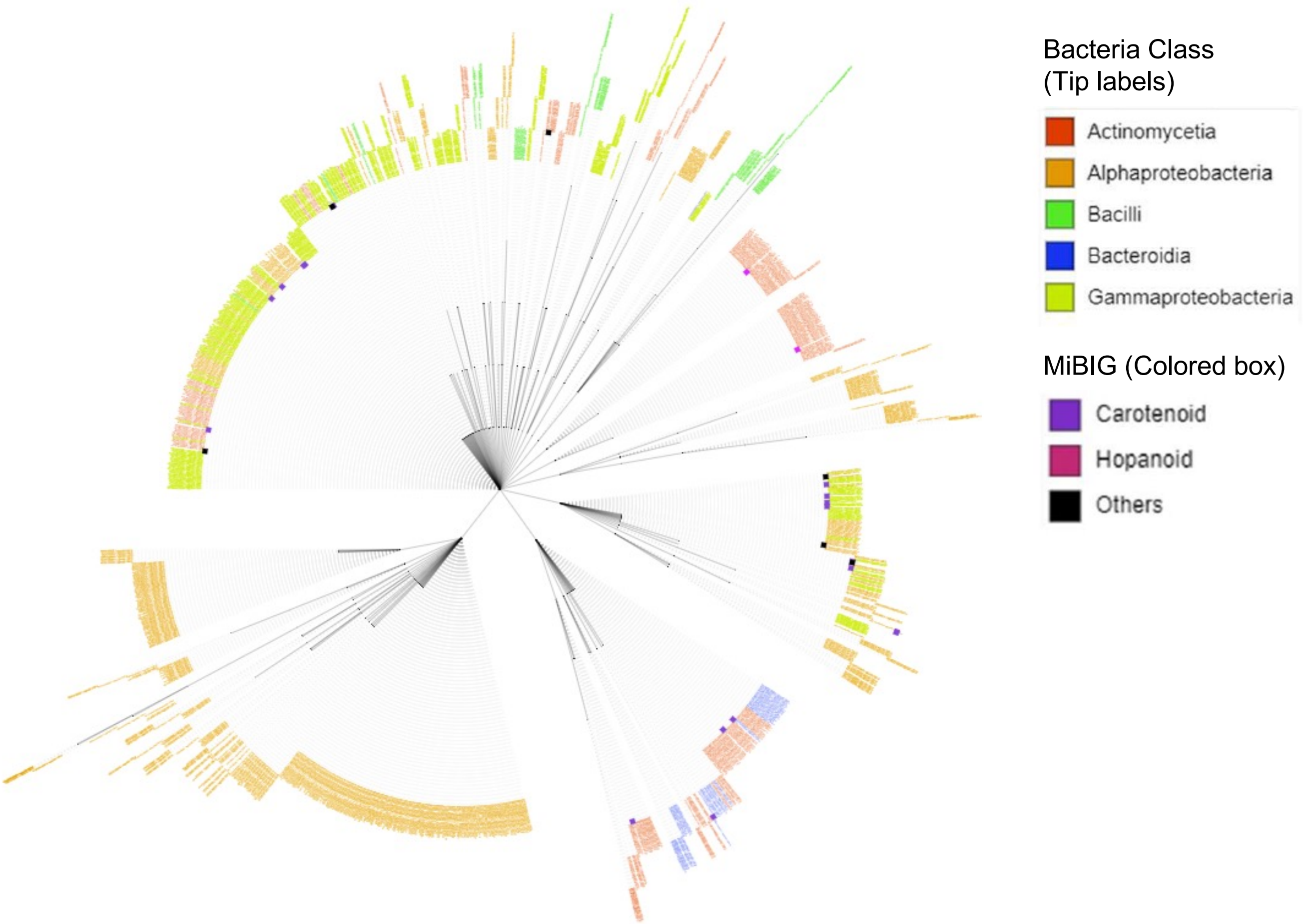

Figure S3. Phylogenetic tree of SQS/PSY domains in plant isolates. The color of tip labels indicates taxonomic information at the class level. The functional role of the domains is indicated in colored squares according to MIBiG.
